# Tendências Recentes de Mortalidade Cardiovascular nas Regiões de Saúde do Estado do Rio de Janeiro e Capital

**DOI:** 10.36660/abc.20190742

**Published:** 2021-04-08

**Authors:** Maria Luiza Garcia Rosa, Claudio Tinoco Mesquita, Lucas Zanetti de Albuquerque, Willian Douglas de Souza Silva, Vinicius de Padua Vieira Alves, Roger Freitas Ramirez Jordan, Ricardo Cardoso de Matos, Ana Luisa Guedes de França e Silva, Erito Marques de Souza

**Affiliations:** 1 Universidade Federal Fluminense Departamento de Epidemiologia e Bioestatística NiteróiRJ Brasil Universidade Federal Fluminense - Departamento de Epidemiologia e Bioestatística, Niterói, RJ - Brasil.; 2 Universidade Federal Fluminense Faculdade de Medicina Departamento de Radiologia NiteróiRJ Brasil Universidade Federal Fluminense Faculdade de Medicina - Departamento de Radiologia, Niterói, RJ - Brasil.; 3 Universidade Federal Fluminense Departamento de Medicina Clínica NiteróiRJ Brasil Universidade Federal Fluminense - Departamento de Medicina Clínica, Niterói, RJ - Brasil.; 4 Universidade Federal Rural do Rio de Janeiro Departamento de Tecnologias e Linguagens Nova IguaçuRJ Brasil Universidade Federal Rural do Rio de Janeiro - Departamento de Tecnologias e Linguagens, Nova Iguaçu, RJ – Brasil.

**Keywords:** Doenças Cardiovasculares/prevenção e controle, Doenças Cerebrovasculares/prevenção e controle, Prevenção de Doenças, Redução, Fatores de Risco, Estilo de Vida, Epidemiologia

## Abstract

**Fundamento:**

A mortalidade por doenças cardiovasculares (DCV) vem mostrando tendência à estabilização em alguns países, incluindo o Brasil e o estado do Rio de Janeiro, após décadas de queda. Não encontramos análises detalhadas dessa tendência para o estado do Rio de Janeiro.

**Objetivo:**

Analisar as tendências da mortalidade prematura e tardia por doenças do aparelho circulatório (DAC), doença isquêmica do coração (DIC) e doença cerebrovascular (DCBV) por sexo nas regiões de saúde do estado do Rio de Janeiro e capital (1996-2016).

**Métodos:**

Dados de óbitos e população foram obtidos no DATASUS/MS. Taxas foram compensadas por códigos mal definidos, corrigidos pelos códigos cardiovasculares mal definidos e ajustadas por sexo e idade pelo método direto. O Joinpoint Trend Analysis Software foi empregado para calcular a variação percentual anual (APC) e variação percentual anual média (AAPC). Foram consideradas para o estudo APC e AAPC significativamente diferentes de zero, calculadas por um teste de student com significância de 5%.

**Resultados:**

A mortalidade por DIC estabilizou ou até aumentou em pelo menos 50% das localidades analisadas (EAPC ≥0). Nas regiões Norte e Noroeste, nenhuma mudança foi observada. Para DCBV, apenas uma região apresentou estabilidade na mortalidade (EAPC próximo a 0). Para as outras regiões, a taxa continuou a diminuir (APC <0) até 2016.

**Conclusão:**

Esses resultados observados no Rio de Janeiro devem se repetir em várias regiões brasileiras e apontam para a necessidade de uma resposta na abordagem dos comportamentos no estilo de vida. Os médicos da atenção primária devem estar familiarizados com a tendência desfavorável da doença isquêmica do coração entre os adultos mais jovens e rastrear ativamente os fatores de risco para DCV, com atenção especial às mulheres.

## Introdução

As doenças cardiovasculares (DCV) são uma das principais causas de morte prematura e incapacidade crônica no mundo e um grande obstáculo ao desenvolvimento humano sustentável, com um número estimado de 422,7 milhões de casos em todo o mundo em 2015, sendo 424.058 destes no Brasil. Em 2011, as Nações Unidas reconheceram formalmente doenças não transmissíveis, incluindo DCV, como uma grande preocupação para a saúde global.

As mudanças sociodemográficas nos últimos 25 anos foram associadas a declínios drásticos nas taxas de mortalidade padronizadas por idade por DCV em regiões com alto índice sociodemográfico, mas apenas uma diminuição gradual no resto do mundo, apesar dos avanços expressivos na capacidade técnica de prevenção e tratamento de DCV.^1^^–^^4^ Os dados do Global Burden of Disease (GBD) de 2015 mostraram redução da mortalidade por DCV padronizada por idade no Brasil e no estado Rio de Janeiro entre 1990 e 2015.^1^^–^^4^

Na última década, estudos internacionais têm demonstrado uma tendência à estabilização dessas taxas.^5^^–^^7^ A mesma tendência foi observada em alguns estados brasileiros, incluindo o Rio de Janeiro.^4^^,^^8^ No entanto, a estabilização dessa taxa de mortalidade não foi analisada por gênero, faixa etária ou regiões do estado, e essa desaceleração no declínio na mortalidade por DCV apresenta um novo desafio para as políticas de saúde em diferentes níveis de cobertura. O objetivo desse estudo é analisar as tendências da mortalidade prematura e tardia de doenças do aparelho circulatório (DAC), doenças isquêmicas do coração (DIC) e doenças cerebrovasculares (DCBV) por sexo na cidade e regiões de saúde do estado do Rio de Janeiro, entre 1996 e 2016, visando avaliar se houve mudanças recentes no padrão de declínio da mortalidade.

## Métodos

Obtivemos dados de mortalidade e população para todos os indivíduos ≥20 anos de idade das regiões de saúde e da capital do estado do Rio de Janeiro entre 1996 e 2016 no *website* do DATASUS/MS.^8^ De acordo com definição do Ministério da Saúde, uma região de saúde consiste em um espaço geográfico contínuo constituído por agrupamentos de municípios limítrofes, delimitado a partir de identidades culturais, econômicas e sociais e de redes de comunicação e infraestrutura de transportes compartilhados, com a finalidade de integrar a organização, o planejamento e a execução de ações de serviços de saúde.^9^

A mortalidade foi categorizada em prematura (30 a 69 anos) e tardia (70 anos ou mais) e foi analisada por sexo e causa para a capital e regiões de saúde do estado do Rio de Janeiro.10 Consideramos os códigos listados no capítulo IX da CID-10 para DAC e, especificamente, os códigos I20-I25 para DIC e I60-I69 para DCBV da CID-10. As taxas de mortalidade bruta e ajustada por sexo e idade (pelo método direto) foram calculadas por 100.000 habitantes, para cada localidade. Atribuímos as mortes por causas mal definidas – códigos do CID-10 RR00-RR99 – às frações observadas nas mortes definidas por DAC, DIC e DCBV (mortes compensadas). Os códigos cardiovasculares mal definidos – I46, I47, I48, I49, I50, I51 e I70 – foram atribuídos à causa DIC, por sexo e categoria de idade.11 Após essas correções, foram estimadas as taxas de mortalidade prematura e tardia ajustadas ao sexo e idade, sempre para cada causa, sexo e localidade. A população de referência foi a população do estado do Rio de Janeiro (censo de 2000), estratificada em sete faixas etárias (20 a 29 anos; 30 a 39 anos; 40 a 49 anos, 50 a 59 anos; 60 a 69 anos; 70 79 anos e 80 anos ou mais) para cada sexo. Essas taxas foram denominadas compensadas e ajustadas.

### Análise Estatística

Para avaliar as tendências, foi empregado o Joinpoint Trend Analysis Software, versão 4.7.0.0.^12^ O modelo de regressão do Joinpoint Poisson calcula a variação percentual anual (APC) a partir da taxa de mortalidade bruta e ajustada. Então, a variação percentual anual média (AAPC) é estimada como o peso médio das APC, em que os períodos são utilizados como peso. Posteriormente, é testado se as APC ou a AAPC são significativamente diferentes de zero por meio de um teste t de Student não pareado com significância de 5%. O número de *joinpoints* foi calculado pelo teste de permutação, utilizando as correções de Bonferroni, em que o nível alfa ajustado foi de 5%. O *software* supõe que o valor padrão do número mínimo de ponto de junção seja 0. Tal método é preferido para produzir resultados mais parcimoniosos.

## Resultados

Em todo o período de 1996 a 2016, a taxa de mortalidade prematura e tardia ajustada à idade para adultos com idade ≥20 anos declinou aproximadamente à metade em todas as regiões do estado do Rio de Janeiro e capital, nos três grupos estudados (DCV, DIC e DCBV), para ambos os sexos quando a AAPC variou de 0,6% a 6,5%. Em geral, a mortalidade prematura para homens foi maior que para as mulheres para as três causas, o que não se repetiu para mortalidade tardia. Os coeficientes para mortalidade tardia foram pelo menos 10 vezes maiores que os da mortalidade prematura, tanto para homens e mulheres, quanto para as três causas (Tabela 1).

**Tabela 1:** Coeficientes ajustados de mortalidade prematura e tardia de doenças cardiovasculares (1996 a 2016) e suas respectivas mudanças percentuais anuais médias | Regiões de saúde do estado do Rio de Janeiro e capital

Regiões de saúde do estado do Rio de Janeiro	Mortalidade prematura																	
	Homens									Mulheres								
		DIC			DCBV			DAC			DIC			DCBV			DAC	
	1996	2016	AAPC	1996	2016	AAPC	1996	2016	-APC	1996	2016	AAPC	1996	2016	AAPC	1996	2016	AAPC
Cidade do Rio de Janeiro	139,24	90,57	-3,0*	82,39	33,76	-5,0*	115,21	67,00	-3,0*	68,00	43,46	-2,8*	59,31	25,33	-4,7*	59,73	35,49	-3,1*
Baía da Ilha Grande	147,07	62,91	-3,2*	105,65	31,04	-5,7*	109,68	46,83	-3,2*	62,72	34,61	-3,5*	69,98	44,44	-5,8*	43,72	27,39	-4,6*
Baixada litorânea	155,30	103,73	-1,3*	86,82	25,67	-5,0*	109,84	86,96	-1,3*	83,22	43,07	-3,3*	93,35	30,13	-5,8*	61,51	30,78	-3,8*
Centro-Sul	257,73	114,03	-3,5*	107,76	34,42	-5,3*	195,31	91,95	-3,5*	98,13	58,19	-3,2*	74,63	43,82	-4,8*	61,88	42,47	-2,3*
Médio-Paraíba	168,61	105,69	-1,9*	105,83	39,62	-5,2*	122,03	86,27	-1,9*	88,17	48,73	-3,2*	87,73	39,23	-5,0*	58,13	37,83	-3,0*
Metropolitana 1	168,86	114,95	-1,5*	124,97	46,78	-4,9*	110,52	84,15	-1,5*	117,10	61,11	-3,0*	129,88	43,76	-5,9*	70,25	45,05	-2,1*
Metropolitana 2	136,54	96,13	-1,2*	93,03	37,91	-5,1*	100,95	80,30	-1,2*	77,24	45,91	-2,0*	80,04	32,95	-5,0*	52,80	36,39	-1,5*
Noroeste	131,49	77,97	-3,1*	125,75	37,59	-5,6*	96,52	57,22	-3,1*	87,25	35,31	-4,0*	53,99	37,14	-4,1*	64,01	28,35	-3,7*
Norte	106,09	80,96	-1,7*	123,00	44,90	-5,1*	71,51	67,02	-1,7*	48,09	42,89	-2,2*	119,25	34,02	-6,0*	30,35	32,48	-1,4*
Serrana	152,97	87,69	-2,6*	105,30	40,81	-4,1*	114,84	69,91	-2,6*	89,91	45,62	-3,5*	90,44	28,63	-4,9*	62,61	35,97	-3,3*

Regiões de saúde do estado do Rio de Janeiro	Mortalidade tardia																	
	Homens									Mulheres								
		DIC			DCBV			DAC			DIC			DCBV			DAC	
	1996	2016	AAPC	1996	2016	AAPC	1996	2016	-APC	1996	2016	AAPC	1996	2016	AAPC	1996	2016	AAPC
Cidade do Rio de Janeiro	1923,61	1082,27	-3,3*	1049,73	521,16	-3,9*	1380,79	785,98	-3,6*	1610,24	807,19	-3,3*	1517,42	733,19	-4,1*	1665,42	838,86	-3,5*
Baía da Ilha Grande	2383,76	1131,36	-3,5*	2309,17	802,26	-6,5*	1544,07	757,86	-2,9*	1940,82	881,83	-3,1*	889,86	322,97	-3,5*	1471,98	590,97	-2,5*
Baixada litorânea	2002,98	1182,20	-2,3*	1667,89	627,68	-3,8*	1193,66	855,53	-1,5*	2497,11	1302,14	-2,7*	742,25	331,48	-3,5*	1495,59	839,08	-1,4*
Centro-Sul	2519,52	1084,25	-4,3*	1527,20	766,98	-3,3*	1549,67	803,94	-3,3*	4040,84	1738,04	-4,1*	717,34	340,08	-4,5*	2080,28	1186,57	-3,9*
Médio-Paraíba	1806,91	1161,65	-2,9*	1541,60	645,67	-3,2*	1103,77	813,33	-2,8*	2655,35	1139,76	-3,9*	676,70	309,13	-3,5*	1485,79	673,55	-3,7*
Metropolitana 1	2120,96	1508,72	-2,1	1661,28	732,68	-4,2*	1122,92	972,22	-1,5	3147,12	1710,64	-2,9*	826,65	295,70	-4,7*	1585,96	1067,85	-2,4*
Metropolitana 2	1776,36	1257,04	-2,1*	1350,81	710,51	-4,2*	1075,85	1020,13	-1,1*	2565,82	1381,72	-2,7*	622,74	253,48	-4,3*	1378,43	1084,99	-0,6
Noroeste	1743,15	971,07	-3,1*	1405,05	713,78	-3,9*	918,39	733,91	-2,0*	2390,88	1114,98	-3,6*	633,83	235,45	-5,0*	1157,58	691,96	-3,3*
Norte	1585,85	832,78	-2,7*	1862,91	854,98	-4,4*	865,80	565,06	-1,7*	2245,17	902,24	-4,0*	821,34	313,52	-4,3*	951,75	606,23	-2,3*
Serrana	2163,82	1178,74	-3,2*	1241,50	761,31	-3,4*	1503,60	834,87	-2,9*	2753,49	1448,35	-3,5*	685,99	352,46	-3,5*	1465,26	970,06	-3,7*

AAPC: variação percentual anual média; APC: variação percentual anual; DIC: doenças isquêmicas do coração; DCBV: doenças cerebrovasculares; DAC: doenças do aparelho circulatório;

* p valor <0,05.

Analisamos as regiões do estado do Rio de Janeiro e a capital no período de 1996 a 2016. Para ambos os sexos, a mortalidade prematura por DIC estabilizou ou até reverteu a tendência em pelo menos 50% das localidades analisadas (APC >0 ou APC ≅0). O mesmo aconteceu com a mortalidade tardia por DIC para as mulheres. Essa mudança ocorreu em diferentes momentos entre 2003 e 2014. Na região metropolitana I, a DIC prematura e tardia para homens e mulheres aumentou a partir 2008 ou 2013. Nas regiões Norte e Noroeste, nenhuma mudança foi observada. Em outras regiões e na capital do Rio de Janeiro, houve mudança na tendência (APC ≥0) para alguns grupos (Tabelas 2 e 3).

**Tabela 2:** Mudança percentual anual (APC) na mortalidade cardiovascular prematura na cidade do Rio de Janeiro (capital) e regiões de saúde do estado do Rio de Janeiro (1996-2016)

Mortalidade prematura					
Grupo de doenças	Região de saúde e capital	Homens		Mulheres	
		Período	APC	Período	APC
DIC	Cidade do Rio de Janeiro	1996-2003	-4,2*	1996-2000	-9,5*
		2003-2008	-0,8	2000-2016	-1,1*
		2008-2014	-3,4*		
		2014-2016	4,2		
	Baía da Ilha Grande			1996-2010	-7,7*
				2010-2016	7,2
	Baixada litorânea			1996-1998	-24,8*
				1998-2004	3,4
				2004-2008	-6,9
				2008-2016	-0,3
	Centro-Sul	1996-2007	-6,8*		
		2007-2016	0,2		
	Médio-Paraíba	1996-2010	-4,2*	1996-2010	-5,3*
		2010-2016	1,7	2010-2016	1,8
	Metropolitana 1	1996-2009	-3,7*	1996-2008	-5,5*
		2009-2016	2,0	2008-2016	0,8
	Metropolitana 2	1996-2009	-2,7*	1996-2009	-4,1*
		2009-2016	0,4	2009-2016	2,0
	Noroeste				
	Norte				
	Serrana				
DCBV	Cidade do Rio de Janeiro				
	Baía da Ilha Grande				
	Baixada litorânea	1996-2005	-2,3		
		2005-2016	-7,2*		
	Centro-Sul				
	Médio-Paraíba				
	Metropolitana 1	1996-2013	-5,5*		
		2013-2016	-1,8		
	Metropolitana 2				
	Noroeste				
	Norte				
	Serrana	1996-2009	-6,3*		
		2009-2016	-0		
DAC	Cidade do Rio de Janeiro	1996-2003	-4,7*	1996-2000	-8,9*
		2003-2008	-1,2	2000-2016	-1,6*
		2008-2014	-5,1*		
		2014-2016	5,8		
	Baía da Ilha Grande				
	Baixada litorânea			1996-1998	-27,3*
				1998-2003	4,6
				2003-2016	-2,7*
	Centro-Sul	1996-2005	-7,5*		
		2005-2016	-0		
	Médio-Paraíba	1996-2012	-3,6*	1996-2010	-5,0*
		2012-2016	5,2	2010-2016	1,7
	Metropolitana 1	1996-2006	-3,0*	1996-2008	-3,7*
		2006-2016	0	2008-2016	0,5
	Metropolitana 2			1996-2007	-3,6*
				2007-2016	1,2
	Noroeste				
	Norte”				
	Serrana				
	Serrana				

APC: mudança percentual anual; DIC: doenças isquêmicas do coração; DCBV: doenças cerebrovasculares; DAC: doenças do aparelho circulatório;

* p valor< 0,05.

Linhas vazias: não foi observada mudança de tendência.

**Tabela 3:** Mudança percentual anual (APC) na mortalidade cardiovascular tardia na cidade do Rio de Janeiro (capital) e regiões de saúde do estado do Rio de Janeiro (1996-2016)

Mortalidade Tardia					
Grupo de doenças	Regiões de saúde e capital	Homens		Mulheres	
		Período	APC	Período	APC
DIC	Cidade do Rio de Janeiro			1996-2011	-5,4*
				2011-2016	3,3
	Baía da Ilha Grande				
	Baixada litorânea				
	Centro-Sul			1996-2010	-6,7*
				2010-2016	2,2
	Médio-Paraíba				
	Metropolitana 1	1996-2001	-7,8*	1996-2008	-6,0*
		2001-2013	-1,9*	2008-2016	1,9
		2013-2016	7,6		
	Metropolitana 2			1996-2010	-5,4*
				2010-2016	3,7
	Noroeste				
	Norte				
	Serrana			1996-2005	-3,1*
				2005-2009	-9,3
				2009-2016	-0,7
DCBV	Cidade do Rio de Janeiro	1996-2004	-1,8*		
		2004-2016	-5,2*		
	Baía da Ilha Grande	1996-1998	-43,4*		
		1998-2016	-1,1		
	Baixada litorânea				
	Centro-Sul				
	Médio-Paraíba				
	Metropolitana 1				
	Metropolitana 2				
	Noroeste				
	Norte				
	Serrana				
DAC	Cidade do Rio de Janeiro			1996-2012	-5,1*
				2012-2016	2,9
	Baía da Ilha Grande				
	Baixada litorânea				
	Centro-Sul				
	Médio-Paraíba				
	Metropolitana 1	1996-2001	-6,2*	1996-2001	-7,8*
		2001-2016	0,1	2001-2016	-0,5
	Metropolitana 2			1996-2006	-2,2*
				2006-2009	-10,4
				2009-2016	6,3*
	Noroeste			1996-2012	-1,2
				2012-2016	-11,2*
	Norte				
	Serrana				
	Serrana				

APC: mudança percentual anual; DIC: doenças isquêmicas do coração; DCBV: doenças cerebrovasculares; DAC: doenças do aparelho circulatório;

* p valor< 0,05.

Linhas vazias: não foi observada mudança de tendência.

Para DCBV, apenas uma região (Serrana) apresentou estabilidade na mortalidade prematura (APC ≅0%). Para as demais regiões, tanto para mortalidade prematura quanto tardia, para homens e mulheres, a taxa continuou a diminuir (APC <0) até 2016 (ver Tabelas 2 e 3).

Em todo o Capítulo IX (CID-10), DAC, observamos uma mudança na tendência da mortalidade prematura em quatro das dez regiões analisadas para homens (Rio de Janeiro capital, Centro-Sul, Médio Paraíba e Metropolitana 1) e em três regiões para mulheres (Médio Paraíba, Metropolitana 1 e Metropolitana 2). Quanto à mortalidade tardia, ocorreram mudanças na tendência da mortalidade em três regiões para mulheres (Rio de Janeiro capital, Metropolitana 1 e Metropolitana 2) e uma para homens (Metropolitana 1). Os anos em que essa mudança ocorreu variaram de 2001 a 2012. A região Metropolitana I apresentou o maior número de mudanças na tendência de mortalidade (ver Tabelas 2 e 3).

É importante ressaltar que, em nenhum dos casos em que houve aumento na mortalidade, essa mudança foi estatisticamente significativa.

## Discussão

A presente análise teve como objetivo identificar mudanças na tendência de diminuição da mortalidade cardiovascular precoce e tardia nas regiões de saúde do estado do Rio de Janeiro e capital, entre 1996 e 2016, de acordo com o sexo.

Pelo menos 50% das localidades mostraram mudanças na mortalidade por DIC, estabilizando ou aumentando a taxa, principalmente para mortalidade prematura em homens e para ambas as mortalidades para mulheres. Para DAC, os resultados foram semelhantes. Esses resultados são particularmente desafiadores, pois a mortalidade prematura impõe um alto ônus social e confirma a evidência de que, no tocante às doenças isquêmicas do coração, as mulheres são subdiagnosticadas e subtratadas, levando ao aumento das complicações e da taxa de mortalidade.^13^

Em uma escala global, a taxa de mortalidade média por DAC ajustada para idade continuamente seguiu um padrão de redução nos anos 1990 e 2000, com o maior declínio entre 2000 e 2005. Isso contou principalmente na redução das taxas de mortalidade por DIC e DCBV, essa última também apresentando a maior queda percentual nos coeficientes de mortalidade prematuro e tardio em todas as regiões de saúde do Rio de Janeiro e capital.^14^ Além disso, regiões de baixa e média renda ao redor do mundo apresentaram uma queda de 13% na taxa de mortalidade ajustada para idade atribuída à DAC, mas com um expressivo aumento de 66% no número total de mortes por essa causa entre 1990 e 2013.^14^ Apesar disso, esse aumento de mortes por DAC atribuídas ao crescimento e envelhecimento populacional foi compensado pela redução em taxas de mortalidade idade-específicas no Brasil, o que aponta para uma possível melhora na qualidade de saúde da população.^15^ No entanto, a distribuição global das DAC é complexa, influenciada por características nacionais e regionais, que resultam em importantes diferenças entre e dentro de regiões, tornando difícil realizar comparações.

Nos EUA, a taxa de declínio para todas as DCV desacelerou substancialmente após 2011.^5^ Argumenta-se que, em parte, o declínio se deve à crescente prevalência de obesidade e diabetes em proporções epidêmicas nos últimos anos, superando os benefícios das políticas de prevenção, os efeitos da prevenção primária e os avanços no tratamento de hipertensão, diabetes e dislipidemia.^1^^,^^3^^,^^16^ No Brasil, embora a cobertura do sistema nacional de vigilância de mortalidade tenha expandido e a qualidade dos certificados de óbito aumentado, com maior proporção de diagnósticos corretos,^17^ não se observa o mesmo para a avaliação da prevalência de fatores de risco. A única fonte de dados seriados sobre fatores de risco cardiovasculares no Brasil é o Vigitel, que cobre apenas capitais. Trata-se de uma pesquisa telefônica anual “nacional”, criticada por alguns por serem autorrelatados. A partir de dados coletados em 2006 a 2016, observamos, para a capital do Rio de Janeiro, aumento na hipertensão autorreferida e obesidade, mas diminuição no tabagismo.^18^

Outros estudos de revisão, como o realizado por Picon et al, estimam taxas de prevalência para hipertensão arterial no Brasil de 28,7% (95% intervalo de confiança, 26,2-31,4) para ambos os sexos em todas as regiões federativas.^19^ Um número considerável de outros estudos sobre a prevalência de fatores de risco cardiovasculares no Brasil pode ser encontrado na literatura, mas, em sua maioria, observam-se limitações metodológicas referentes à cobertura espacial e ausência de exames laboratoriais confirmatórios.^20^

Nas regiões do Rio de Janeiro, foram observadas mudanças nas tendências de mortalidade por DIC e DAC, com estabilização ou até mesmo aumento das taxas entre 2004 e 2014. Descreve-se que as primeiras evidências de uma estabilização e sinais precoces de aumento entre jovens adultos nos EUA, embora sem significância estatística, foram publicadas pela primeira vez em 2007.^21^ Muitos fatores podem se relacionar com essa tendência regional observada no Rio de Janeiro, e dados que mostram melhora na qualidade de saúde e de vida tornam difícil compreender a razão para essa tendência de achatamento observada.^22^ A cobertura do sistema de saúde suplementar no estado do Rio de Janeiro em 2013 era de 33,5%, com aumento anual de 0,78% entre 2004 e 2013. Correlações lineares inversas com taxas de mortalidade por DAC e DCBV foram observadas, o que deve ser questionado pela possibilidade de outros fatores simultâneos – como o aumento no índice de desenvolvimento humano municipal em todas as unidades federativas entre 2000 e 2010 – também terem afetado essas taxas.^21^

Conforme discutido por Soares et al. em dois trabalhos diferentes, melhoras socioeconômicas precederam o declínio na mortalidade por DCV.^23^^,^^24^ A redução nas taxas de mortalidade, principalmente por DIC no estado do Rio de Janeiro nas últimas décadas, foi precedida por um aumento no índice de desenvolvimento humano (IDH).^23^ Apenas poucos municípios do estado do Rio de Janeiro tiveram queda no IDH entre 1970 e 1991. Alguns deles estão localizados nas regiões norte e nordeste, onde a mortalidade por DIC está caindo continuamente. Essas observações permitem levantar a hipótese de que esses municípios estão se beneficiando das melhoras socioeconômicas que ocorreram anteriormente em regiões onde há uma inversão na tendência da mortalidade, chamando a atenção para a Metropolitana I.

Nos EUA, o declínio em toda a mortalidade por DCV desacelerou substancialmente, incluindo a DCBV, o que não ocorreu nas regiões de saúde e capital do Rio de Janeiro.^2^ Isso pode ser explicado pelo atraso na implementação de medidas primárias de prevenção de acidente vascular encefálico (AVE) isquêmico (estatinas, ácido acetilsalicílico, terapia antitrombótica) em comparação com países desenvolvidos. Em escala nacional, o achatamento do padrão das taxas de mortalidade por DAC observados nos anos recentes serve como sinal de alerta para a necessidade de monitoramento contínuo e a possibilidade da emergência de contratendências significativas G.^2^ O sistema de saúde público, com programas como a estratégia de saúde da família e programa da farmácia popular, tem importância indubitável no controle da hipertensão e outros fatores de risco cardiovascular.^25^^,^^26^ Campanhas nacionais efetivas antitabaco, planos nacionais de prevenção e controle da obesidade e outras políticas com objetivo de monitorar e reduzir fatores de risco fizeram parte da história recente do desenvolvimento do sistema de saúde público brasileiro.^27^^–^^29^ É crucial entender os mecanismos que podem estar prejudicando sua eficiência.

### Limitações

O presente estudo tem algumas limitações. Uma delas é a qualidade das declarações de óbito, que não é uniforme entre os municípios e tem variado ao longo do tempo, o que pode ter afetado as tendências observadas. A segunda limitação é a existência de municípios que compõem as regiões de saúde com pequenas populações, o que leva a grandes oscilações na ocorrência de eventos pouco frequentes, como a morte. A terceira limitação é a ausência de informações sobre a prevalência dos fatores de risco cardiovascular para as localidades analisadas.

## Conclusão

As tendências adversas de mortalidade por DIC e DAC em homens adultos jovens e mulheres nas duas faixas etárias foram observadas em 50% das regiões de saúde no estado do Rio de Janeiro. Esses resultados observados no Rio de Janeiro devem se repetir em várias regiões brasileiras, e apontam para a necessidade de uma resposta na abordagem dos comportamentos no estilo de vida. Os médicos da atenção primária devem estar familiarizados com a tendência desfavorável da doença isquêmica do coração entre os adultos mais jovens e rastrear ativamente os fatores de risco para DCV, com atenção especial às mulheres.
