# Supplementary material for: Tendências Recentes de Mortalidade Cardiovascular nas Regiões de Saúde do Estado do Rio de Janeiro e Capital
Source: Arq Bras Cardiol. 2021 Apr 8;116(4):763–71. [Article in Portuguese] doi: 10.36660/abc.20190742 (PMC8121409; doi:10.36660/abc.20190742)
Supplement: Translation

# Translation: Recent Trends in Cardiovascular Mortality in Rio de Janeiro State Health Regions and Capital

**Authors:** Maria Luiza Garcia Rosa, Claudio Tinoco Mesquita, Lucas Zanetti de Albuquerque, Willian Douglas de Souza Silva, Vinicius de Padua Vieira Alves, Roger Freitas Ramirez Jordan, Ricardo Cardoso de Matos, Ana Luisa Guedes de França e Silva, Erito Marques de Souza

**Affiliations:** 1 NiteróiRJ Brazil Universidade Federal Fluminense - Departamento de Epidemiologia e Bioestatística, Niterói, RJ - Brazil.; 2 NiteróiRJ Brazil Universidade Federal Fluminense Faculdade de Medicina - Departamento de Radiologia, Niterói, RJ - Brazil.; 3 NiteróiRJ Brazil Universidade Federal Fluminense - Departamento de Medicina Clínica, Niterói, RJ - Brazil.; 4 Nova IguaçuRJ Brazil Universidade Federal Rural do Rio de Janeiro - Departamento de Tecnologias e Linguagens, Nova Iguaçu, RJ – Brazil.

**Keywords:** Cardiovascular Diseases/prevention and control, Cerebrovascular Diseases/prevention and control, Disease Prevention, Risk Factors, Life Style, Epidemiology

## Abstract

**Background:**

Cardiovascular disease (CVD) mortality, after several decades of decrease, has shown a tendency towards the stabilization in some countries, including Brazil and Rio de Janeiro state. This new tendency was not further analyzed by gender, age group and region of the Rio de Janeiro state.

**Objective:**

To analyze the trends of premature and late mortality from CVD, ischemic heart disease (IHD) and cerebrovascular disease (CBVD) by gender in the city of Rio de Janeiro (capital) and the health regions of Rio de Janeiro state (from 1996 to 2016.

**Methods:**

Data on deaths and the population were obtained from DATASUS/MS. The rates were compensated by ill-defined codes, corrected by Ill-Defined Cardiovascular codes and gender and age-adjusted by the direct method (reference population – population of the state of Rio de Janeiro - 2000 census). The Joinpoint Trend Analysis Software was employed.

**Results:**

IHD mortality stabilized or even increased for at least 50% of the analyzed areas (EAPC≥0). No change was observed. in the “North” and “Northwest” regions For CBVD, just one region showed stability regarding mortality (EAPC close to 0). For the other regions, the rate continued to decrease (APC<0) until 2016.

**Conclusion:**

These results observed in Rio de Janeiro are possibly appropriate to various Brazilian regions and demonstrate that a serious public health response is needed to address lifestyle behaviors. Primary care physicians should also be familiar with the unfavorable tendency in coronary heart disease among younger adults in recent years and actively screen for risk factors for cardiovascular disease, paying special attention to women.

## Introduction

Cardiovascular diseases (CVDs) are a leading cause of premature death and chronic disability worldwide and a major obstacle to sustainable human development, with an estimated 422.7 million cases occurring worldwide in 2015 (424,058 of these cases in Brazil). In 2011, the United Nations formally recognized noncommunicable diseases, including CVDs, as a major concern for global health.

Sociodemographic changes over the past 25 years have been associated with dramatic declines in age-standardized rates of CVD mortality in regions with high sociodemographic Index (SDI), but only a gradual decrease in the rest of the globe despite impressive advances in technical capacity for preventing and treating CVD.^1^^–^^4^ Data from the 2015 Global Burden of Disease (GBD) showed a reduction in age-related CVD mortality in Brazil and in Rio de Janeiro state between 1990 and 2015.^1^^–^^4^

In the last decade, international studies have observed a tendency towards the stabilization of these CVD mortality rates.^5^^–^^7^ The same trend was observed in some Brazilian states, including in Rio de Janeiro.^4^^,^^8^ However, this mortality rate stabilization in Rio de Janeiro state was not further analyzed by gender, age group nor between state regions and this decelerating decline in CVD mortality poses a new challenge for health policies at different levels of coverage. The objective of this study is to analyze the trends of the premature and late mortality from CVD, ischemic heart disease (IHD) and cerebrovascular disease (CBVD) by gender, for the capital city and health regions of Rio de Janeiro state, between 1996 and 2016 to determine whether there have been any recent changes in the mortality decline pattern.

## Methods

We obtained data on mortality and population for all individuals aged ≥20 years in the Rio de Janeiro state capital and health regions, between 1996 and 2016 from the DATASUS website.^8^ According to the Brazilian Ministry of Health, a health region consists of a continuous geographical space consisting of groups of municipalities with defined limits, delimited based on cultural, economic and social identities and shared communication networks and transport infrastructure, with the intent of integrating organization, planning and implementation of health actions and services.^9^

Mortality was categorized as premature (30-69 years) and late (70 years or more) and was analyzed by gender and cause for the state capital city of Rio de Janeiro and regions of the state.^10^ We considered all codes listed in chapter IX of ICD-10 for CVD and, specifically, the codes I20-I25 for IHD and I60-I69 for CBVD of ICD-10. The crude and gender- and age-adjusted mortality rates (by the direct method) were calculated per 100,000 individuals, for each locality. We assigned the deaths from ill-defined causes (IDC)– ICD codes RR00-RR99 – to the fractions observed in the defined deaths from CVD, IHD and CBVD (compensated deaths). The Ill-Defined Cardiovascular Codes – I46, I47, I48, I49, I50, I51 and I70 were assigned to the IHD cause, by gender and age category.^11^ After those corrections, age-adjusted premature and late mortality rates were estimated, always for each cause, gender and locality. The reference population was the state of Rio de Janeiro population (2000 census), stratified into seven age groups (20 to 29 years; 30 to 39 years; 40 to 49 years old, 50 to 59 years old; 60 to 69 years old; 70 to 79 years old; and 80 years or older) in each gender. Those rates were denominated as compensated and adjusted.

### Statistical Analysis

To evaluate the trends, the Joinpoint Trend Analysis Software version 4.7.0.0 was employed.^12^ The Joinpoint Poisson regression model estimates the annual percentage change (APC) of crude and adjusted death rates. Then, the average annual percentage change (AAPC) is estimated as a weight average of the estimated APC using the period as the weight. Subsequently, it is tested whether the APC or AAPC are significantly different from zero using the unpaired Student's t-test, with alpha = 5%. The number of jointpoints was calculated by permutation test, using Bonferroni corrections, where the adjusted alpha level was 5%. The software assumes that the default value of the minimum number of jointpoints is 0. This method is the preferred one to produce more parsimonious results.

## Results

We analyzed the regions of Rio de Janeiro state and the Rio de Janeiro city Capital from 1996 to 2016. During the entire period, the age-adjusted premature and late mortality rate for adults ≥20 years old plummeted to around 50% for all regions of the state of Rio de Janeiro and capital city in the three studied groups (CVD, IHD and CBVD), for both genders, when the AAPC ranged from −0.6% to −6.5%. In general, the premature mortality for men was higher than that for women for the three causes, which was not necessarily the same for late mortality. The late mortality coefficients were at least 10 times higher than those for premature mortality for both genders and for the three causes of mortality (Table 1).

**Table 1:** Premature and Late Adjusted Mortality coefficients of Cardiovascular Diseases (1996 – 2016) and respectives annual average percentual change (AAPC). Regions of Health of Rio de Janeiro State

Regions of Health of Rio de Janeiro State	Premature Mortality																	
	Men									Women								
		IHD			CBVD			CVD			IHD			CBVD			CVD	
	1996	2016	AAPC	1996	2016	AAPC	1996	2016	AAPC	1996	2016	AAPC	1996	2016	AAPC	1996	2016	AAPC
Rio de Janeiro City	139.24	90.57	-3,0*	82.39	33.76	-5,0*	115.21	67.00	-3,0*	68.00	43.46	-2,8*	59.31	25.33	-4,7*	59.73	35.49	-3,1*
“Baia de Ilha Grande”	147.07	62.91	-3,2*	105.65	31.04	-5,7*	109.68	46.83	-3,2*	62.72	34.61	-3,5*	69.98	44.44	-5,8*	43.72	27.39	-4,6*
“Baixada Litoranea”	155.30	103.73	-1,3*	86.82	25.67	-5,0*	109.84	86.96	-1,3*	83.22	43.07	-3,3*	93.35	30.13	-5,8*	61.51	30.78	-3,8*
“Centro Sul”	257.73	114.03	-3,5*	107.76	34.42	-5,3*	195.31	91.95	-3,5*	98.13	58.19	-3,2*	74.63	43.82	-4,8*	61.88	42.47	-2,3*
“Medio Paraiba”	168.61	105.69	-1,9*	105.83	39.62	-5,2*	122.03	86.27	-1,9*	88.17	48.73	-3,2*	87.73	39.23	-5,0*	58.13	37.83	-3,0*
“Metropolitana 1”	168.86	114.95	-1,5*	124.97	46.78	-4,9*	110.52	84.15	-1,5*	117.10	61.11	-3,0*	129.88	43.76	-5,9*	70.25	45.05	-2,1*
“Metropolitana 2”	136.54	96.13	-1,2*	93.03	37.91	-5,1*	100.95	80.30	-1,2*	77.24	45.91	-2,0*	80.04	32.95	-5,0*	52.80	36.39	-1,5*
“Noroeste”	131.49	77.97	-3,1*	125.75	37.59	-5,6*	96.52	57.22	-3,1*	87.25	35.31	-4,0*	53.99	37.14	-4,1*	64.01	28.35	-3,7*
“Norte”	106.09	80.96	-1,7*	123.00	44.90	-5,1*	71.51	67.02	-1,7*	48.09	42.89	-2,2*	119.25	34.02	-6,0*	30.35	32.48	-1,4*
“Serrana”	152.97	87.69	-2,6*	105.30	40.81	-4,1*	114.84	69.91	-2,6*	89.91	45.62	-3,5*	90.44	28.63	-4,9*	62.61	35.97	-3,3*

Regions of Health of Rio de Janeiro State	Late Mortality																	
	Men									Women								
		IHD			CBVD			CVD			IHD			CBVD			CVD	
	1996	2016	AAPC	1996	2016	AAPC	1996	2016	AAPC	1996	2016	AAPC	1996	2016	AAPC	1996	2016	AAPC
Rio de Janeiro City	1923.61	1082.27	-3,3*	1049.73	521.16	-3,9*	1380.79	785.98	-3,6*	1610.24	807.19	-3,3*	1517.42	733.19	-4,1*	1665.42	838.86	-3,5*
“Baia de Ilha Grande”	2383.76	1131.36	-3,5*	2309.17	802.26	-6,5*	1544.07	757.86	-2,9*	1940.82	881.83	-3,1*	889.86	322.97	-3,5*	1471.98	590.97	-2,5*
“Baixada Litoranea”	2002.98	1182.20	-2,3*	1667.89	627.68	-3,8*	1193.66	855.53	-1,5*	2497.11	1302.14	-2,7*	742.25	331.48	-3,5*	1495.59	839.08	-1,4*
“Centro Sul”	2519.52	1084.25	-4,3*	1527.20	766.98	-3,3*	1549.67	803.94	-3,3*	4040.84	1738.04	-4,1*	717.34	340.08	-4,5*	2080.28	1186.57	-3,9*
“Medio Paraiba”	1806.91	1161.65	-2,9*	1541.60	645.67	-3,2*	1103.77	813.33	-2,8*	2655.35	1139.76	-3,9*	676.70	309.13	-3,5*	1485.79	673.55	-3,7*
“Metropolitana 1”	2120.96	1508.72	-2,1	1661.28	732.68	-4,2*	1122.92	972.22	-1,5	3147.12	1710.64	-2,9*	826.65	295.70	-4,7*	1585.96	1067.85	-2,4*
“Metropolitana 2”	1776.36	1257.04	-2,1*	1350.81	710.51	-4,2*	1075.85	1020.13	-1,1*	2565.82	1381.72	-2,7*	622.74	253.48	-4,3*	1378.43	1084.99	-0,6
“Noroeste”	1743.15	971.07	-3,1*	1405.05	713.78	-3,9*	918.39	733.91	-2,0*	2390.88	1114.98	-3,6*	633.83	235.45	-5,0*	1157.58	691.96	-3,3*
“Norte”	1585.85	832.78	-2,7*	1862.91	854.98	-4,4*	865.80	565.06	-1,7*	2245.17	902.24	-4,0*	821.34	313.52	-4,3*	951.75	606.23	-2,3*
“Serrana”	2163.82	1178.74	-3,2*	1241.50	761.31	-3,4*	1503.60	834.87	-2,9*	2753.49	1448.35	-3,5*	685.99	352.46	-3,5*	1465.26	970.06	-3,7*

IHD: ischemic heart disease; CBVD: cerebrovascular disease; CVD: cardiovascular diseases;

* Pvalue<0.05.

From 1996 to 2016, for both genders, the premature IHD mortality stabilized or even reversed the trend for at least 50% of the analyzed areas (APC>0 or APC≅0%). The same happened for late IHD mortality for women. This change occurred in different moments between 2003 and 2014. In the “Metropolitan I region” the premature and late mortality from IHD for both men and women, has increased since 2008 or 2013. In the “North” and “Northwest” regions, no change was observed. In other regions and in Rio de Janeiro state capital, there was change in the tendency (APC>0 or APC≅0%) for some groups (Tables 2 and 3).

**Table 2:** Annual percent change (APC) in cardiovascular premature mortality for Rio de Janeiro capital and regions of Rio de Janeiro state. 1996-2016

Premature Mortality					
Disease group	Health region	Men		Women	
		Period	APC	Period	APC
IHD	Rio de Janeiro City	1996-2003	-4,2*	1996-2000	-9,5*
		2003-2008	-0,8	2000-2016	-1,1*
		2008-2014	-3,4*		
		2014-2016	4,2		
	“Baia de Ilha Grande”			1996-2010	-7,7*
				2010-2016	7,2
	“Baixada Litoranea”			1996-1998	-24,8*
				1998-2004	3,4
				2004-2008	-6,9
				2008-2016	-0,3
	“Centro Sul”	1996-2007	-6,8*		
		2007-2016	0,2		
	“Medio Paraiba”	1996-2010	-4,2*	1996-2010	-5,3*
		2010-2016	1,7	2010-2016	1,8
	“Metropolitana 1”	1996-2009	-3,7*	1996-2008	-5,5*
		2009-2016	2,0	2008-2016	0,8
	“Metropolitana 2”	1996-2009	-2,7*	1996-2009	-4,1*
		2009-2016	0,4	2009-2016	2,0
	“Noroeste”				
	“Norte”				
	“Serrana”				
CBVD	Rio de Janeiro City				
	“Baia de Ilha Grande”				
	“Baixada Litoranea”	1996-2005	-2,3		
		2005-2016	-7,2*		
	“Centro Sul”				
	“Medio Paraiba”				
	“Metropolitana 1”	1996-2013	-5,5*		
		2013-2016	-1,8		
	“Metropolitana 2”				
	“Noroeste”				
	“Norte”				
	“Serrana”	1996-2009	-6,3*		
		2009-2016	-0		
CVD	Rio de Janeiro City	1996-2003	-4,7*	1996-2000	-8,9*
		2003-2008	-1,2	2000-2016	-1,6*
		2008-2014	-5,1*		
		2014-2016	5,8		
	“Baia de Ilha Grande”				
	“Baixada Litoranea”			1996-1998	-27,3*
				1998-2003	4.6
				2003-2016	-2,7*
	“Centro Sul”	1996-2005	-7,5*		
		2005-2016	-0		
	“Medio Paraiba”	1996-2012	-3,6*	1996-2010	-5,0*
		2012-2016	5,2	2010-2016	1.7
	“Metropolitana 1”	1996-2006	-3,0*	1996-2008	-3,7*
		2006-2016	0	2008-2016	0,5
	“Metropolitana 2”			1996-2007	-3,6*
				2007-2016	1,2
	“Noroeste”				
	“Norte”				
	“Serrana”				
	“Serrana”				

APC: annual percent change; IHD: ischemic heart disease; CBVD: cerebrovascular disease; CVD: cardiovascular diseases;

* Pvalue<0.05.

**Table 3:** Annual percent change (APC) in cardiovascular late mortality for Rio de Janeiro capital and regions of Rio de Janeiro state. 1996-2016

Late mortality					
Disease group	Health region	Men		Women	
		Period	APC	Period	APC
IHD	Rio de Janeiro City			1996-2011	-5,4*
				2011-2016	3,3
	“Baia de Ilha Grande”				
	“Baixada Litoranea”				
	“Centro Sul”			1996-2010	-6,7*
				2010-2016	2,2
	“Medio Paraiba”				
	“Metropolitana 1”	1996-2001	-7,8*	1996-2008	-6,0*
		2001-2013	-1,9*	2008-2016	1,9
		2013-2016	7,6		
	“Metropolitana 2”			1996-2010	-5,4*
				2010-2016	3,7
	“Noroeste”				
	“Norte”				
	“Serrana”			1996-2005	-3,1*
				2005-2009	-9,3
				2009-2016	-0,7
CBVD	Rio de Janeiro City	1996-2004	-1,8*		
		2004-2016	-5,2*		
	“Baia de Ilha Grande”	1996-1998	-43,4*		
		1998-2016	-1,1		
	“Baixada Litoranea”				
	“Centro Sul”				
	“Medio Paraiba”				
	“Metropolitana 1”				
	“Metropolitana 2”				
	“Noroeste”				
	“Norte”				
	“Serrana”				
CVD	Rio de Janeiro City			1996-2012	-5,1*
				2012-2016	2.9
	“Baia de Ilha Grande”				
	“Baixada Litoranea”				
	“Centro Sul”				
	“Medio Paraiba”				
	“Metropolitana 1”	1996-2001	-6,2*	1996-2001	-7,8*
		2001-2016	0,1	2001-2016	-0,5
	“Metropolitana 2”			1996-2006	-2,2*
				2006-2009	-10,4
				2009-2016	6,3*
	“Noroeste”			1996-2012	-1,2
				2012-2016	-11,2*
	“Norte”				
	“Serrana”				
	“Serrana”				

APC: annual percent change; IHD: ischemic heart disease; CBVD: cerebrovascular disease; CVD: cardiovascular diseases;

* Pvalue<0.05.

For CBVD, only one region showed stability in premature mortality (APC>0 or APC≅0%). For the other regions, either for premature or late mortality, for both men and women, the rate continued to increase until 2016 (Tables 2 and 3).

For the entire chapter IX (ICD X), CVD, we observed a change in the trend in premature mortality in four of the ten analyzed regions for men and in three regions for late mortality in women. The years during which these changes occurred went from 2001 to 2014. Metropolitan region 1 showed the largest number of changes in the mortality trend (Tables 2 and 3). In Rio de Janeiro city Capital, a decrease in mortality was observed for men regarding late mortality and for both genders regarding premature mortality. A decrease was observed for late mortality in women until 2011, followed by a reverse in rates, with a small increase until 2016. (Figure 1)

It is important to underscore that none of the cases which showed an increase in mortality had a statistically significant change.

## Discussion

The present analysis aimed to identify changes in the trend of decreasing early and late cardiovascular mortality in the health regions of Rio de Janeiro state and city capital, between 1996 and 2016, according to gender.

At least 50% of the areas showed changes in IHD mortality, either stabilizing or increasing the rate, mostly due to premature mortality in men and for both types of mortality for women. The results were similar for CVD. These results are particularly challenging as premature mortality has a high social impact and confirms the evidence that there is a lack of identification of IHD, as well as undertreatment and undertesting of women as causes of higher mortality rates and increased morbid complications in the female gender.^13^

In a global scale, the average age-standardized CVD death rate continuously followed a pattern of reduction in the decades of 1990s and 2000s, with the greatest decline occurring between 2000 and 2005. This mostly counted on the reduction of mortality rates due to IHD and CBVD, with the latter also showing the most prominent percentage reductions in premature and late death coefficients in all Rio de Janeiro health regions and capital city.^14^ Also, low and middle-income regions around the world have shown a decrease of 13% in age-standardized rates of death attributable to CVD but with a significant 66% growth in the number of deaths between 1990 and 2013.^14^ Despite that, this increase in CVD deaths due to population growth and aging was compensated by a reduction in age-specific death rates in Brazil, indicating possible improvements in the population's health quality.^15^ However, the global distribution of CVD is a complex one, influenced by national and regional characteristics, which results in significant differences between and within regions, making comparisons difficult to accomplish.

In the USA, the rate of decline in all cardiovascular diseases substantially decelerated after 2011.^5^ It is argued that, in part, the decline is due to the increasing prevalence of obesity and diabetes at epidemic proportions in recent years, overcoming the benefits of prevention policies, the effects of primary prevention and advances in the treatment of hypertension, diabetes and dyslipidemia.^1^^,^^3^^,^^16^ In Brazil, although the coverage of the national mortality surveillance system has been expanded and the quality of death certification has improved, with a higher proportion of correct diagnoses,^17^ the same is not observed for the evaluation of risk factor prevalence. The only source of serial cardiovascular risk factors in Brazil is the “Vigitel”, which covers only the state capitals. This national and annual phone survey is criticized by some for being self-reported. Based on data collection carried out in 2006 and 2016, we observed that there was an increase in self-reported hypertension and obesity for Rio de Janeiro state capital, whereas there was a decrease in self-reported smoking status.^18^

Other review studies, such as the one performed by Picon et al., has estimated prevalence rates for hypertension in Brazil of 28.7% (95% confidence interval [CI], 26.2–31.4), for both genders in all federation units.^19^ A considerable number of other studies about the prevalence of CVD risk factors in our country can be found in the literature, but most have methodological limitations related to spatial coverage or absence of confirmatory laboratory examinations.^20^

In all Rio de Janeiro regions, the changes in IHD or CVD mortality trends, with stabilization or even increase of the rates, were observed between 2004 and 2014. In fact, evidence that cardiovascular mortality rates for young adults in the USA could be stabilizing or even showing early nonsignificant signs of an increase was first published in 2007.^21^ Many factors can be related to this observed regional trend, and data showing health and quality of life improvements makes it harder to understand the reason this flattening trend was observed. Supplementary health coverage in the state of Rio de Janeiro in 2013 was 33.5%, with an annual increase of 0.78% between 2004 and 2013. Inverse linear correlations with mortality rates due to CVD and CBVD were observed, which must be questioned due to possibility of other facts, since the simultaneous increase in municipal human development index in all federal units between 2000 and 2010 can also have affected these rates.^21^

As discussed by Soares et al. in two different articles, socioeconomic improvements preceded the decline in mortality due to cardiovascular diseases.^23^^,^^24^ The reduction in the mortality rates, particularly from IHD in the Rio de Janeiro state in the past decades, was preceded by an increase in the Human Development Index (HDI).^23^ Only a few municipalities in the Rio de Janeiro state showed a decrease in the HDI between 1970 and 1991. Some of them are located in the “north” and “northwest” regions, where the mortality from IHD is continuously declining. These observations allow us to raise the hypothesis that these municipalities are benefiting from socioeconomic improvements that occurred earlier in the regions where there was an inversion in the mortality trend, drawing attention to the “Metropolitan I” region.

In the USA, the decline in all CVD mortality has decelerated substantially, including CBVD, which did not occur in Rio de Janeiro health regions and capital city.^2^ This may be explained by the delay in the implementation of primary prevention measures for ischemic stroke (statins, aspirin, antithrombotic therapy) compared to developed countries. At the national scale, the flattening pattern of mortality rate due to CVD observed in recent years serve as a warning signal to the need for continuing monitoring of trends and the possibility of emergence of significant countertrends.^2^ The public health network, with programs such as Family Health Strategy and Popular Pharmacy Program, are undoubtedly important in controlling hypertension and other cardiovascular risk factors.^25^^,^^26^ Effective national anti-tobacco and obesity prevention campaigns and national control plans and other policies with the objective to monitor and reduce risk factors were part of the recent history of Brazil national public health system development.^27^^–^^29^^8^ Understanding the mechanisms that may be hindering its effectiveness is a crucial step.

### Limitations

The present study has some limitations. One of them is the quality of death certificates, which is different for the municipalities that comprise the regions, and varied over time, affecting the observed trends. The second limitation is the existence of municipalities with small populations that comprise the regions, which leads to large oscillations in the occurrence of infrequent events, such as death. The third limitation is the lack of information about the prevalence of cardiovascular risk factors for the analyzed areas.

## Conclusion

The adverse IHD and CVD mortality trends observed in young adult men and in young and older women were observed in 50% of the Rio de Janeiro regions. These results are possibly appropriate to several regions of the country and demonstrates that a serious public health response is needed to address lifestyle behaviors. Primary care physicians should also be familiar with the unfavorable tendency in coronary heart disease in younger adults in recent years and actively screen for risk factors for cardiovascular disease, paying special attention to women.
